# Melatonin accelerates the developmental competence and telomere elongation in ovine SCNT embryos

**DOI:** 10.1371/journal.pone.0267598

**Published:** 2022-07-21

**Authors:** Parisa Nadri, Saeid Ansari-Mahyari, Farnoosh Jafarpour, Amir Hossein Mahdavi, Nima Tanhaei Vash, Liana Lachinani, Kianoush Dormiani, Mohammad Hossein Nasr-Esfahani

**Affiliations:** 1 Department of Animal Science, College of Agriculture, Isfahan University of Technology, Isfahan, Iran; 2 Department of Animal Biotechnology, Reproductive Biomedicine Research Center, Royan Institute for Biotechnology, ACECR, Isfahan, Iran; 3 Department of Animal Biotechnology, Cell Science Research Center, Royan Institute for Biotechnology, ACECR, Isfahan, Iran; Justus Liebig Universitat Giessen, GERMANY

## Abstract

SCNT embryos suffer from poor developmental competence (both *in vitro* and *in vivo*) due to various defects such as oxidative stress, incomplete epigenetic reprogramming, and flaws in telomere rejuvenation. It is very promising to ameliorate all these defects in SCNT embryos by supplementing the culture medium with a single compound. It has been demonstrated that melatonin, as a multitasking molecule, can improve the development of SCNT embryos, but its function during ovine SCNT embryos is unclear. We observed that supplementation of embryonic culture medium with 10 nM melatonin for 7 days accelerated the rate of blastocyst formation in ovine SCNT embryos. In addition, the quality of blastocysts increased in the melatonin-treated group compared with the SCNT control groups in terms of ICM, TE, total cell number, and mRNA expression of NANOG. Mechanistic studies in this study revealed that the melatonin-treated group had significantly lower ROS level, apoptotic cell ratio, and mRNA expression of CASPASE-3 and BAX/BCL2 ratio. In addition, melatonin promotes mitochondrial membrane potential and autophagy status (higher number of LC3B dots). Our results indicate that melatonin decreased the global level of 5mC and increased the level of H3K9ac in the treated blastocyst group compared with the blastocysts in the control group. More importantly, we demonstrated for the first time that melatonin treatment promoted telomere elongation in ovine SCNT embryos. This result offers the possibility of better development of ovine SCNT embryos after implantation. We concluded that melatonin can accelerate the reprogramming of telomere length in sheep SCNT embryos, in addition to its various beneficial effects such as increasing antioxidant capacity, reducing DNA damage, and improving the quality of derived blastocysts, all of which led to a higher *in vitro* development rate.

## Introduction

The oocyte, as a unique cell in both structure and function, is capable of supporting the full development of embryos from somatic cell nuclear transfer (SCNT). After the birth of Dolly in 1997, the SCNT technique has been applied to more than 20 animal species for the purpose of reproductive cloning [1]. Despite the successful results of SCNT technique, the low efficiency (1–2%) of this technique is no longer a matter of debate, which limits its application [2]. Several factors such as suboptimal culture conditions, oxidative stress, autophagy status, telomere length, epigenetic barriers in donor somatic cells, etc. are responsible for the low success rate of the SCNT technique [3–5].

The physiological level of intracellular reactive oxygen species (ROS) is an essential factor for maintaining the proper function of male and female reproductive systems [6, 7]. Supraphysiological level of ROS lead to oxidative stress, which impairs the ability of sperm, oocytes, and resulting embryos to develop [8–10]. Several studies have shown that elevated levels of ROS under *in vitro* conditions can contribute to embryonic apoptosis, mitochondrial dysfunction, and subsequent developmental arrest [10–12]. Moreover, oxidative stress and DNA damage have been shown to be higher in SCNT embryos than in *in vitro* fertilized (IVF) embryos due to some specific procedures such as enucleation, cell attachment, electrical fusion and chemical activation [4, 13]. One of the most effective strategies to improve cellular antioxidant capacity to overcome the excessive production of ROS is the addition of exogenous antioxidants to the culture media. In this regard, numerous studies have reported that supplementing the maturation or/and culture medium with exogenous antioxidants accelerates embryonic development [14].

Scientists have demonstrated the role of autophagy in several developmental features, including pre- and post-embryonic development and placental and fetal growth. Tsukamoto and colleagues have shown that autophagy can be used as a valuable marker for selecting high-quality embryos for further development [15, 16]. Limited studies have shown that autophagy is not induced in SCNT embryos compared to IVF embryos. Tsukamoto and colleagues have also shown that pharmacological activation of autophagy in SCNT-reconstituted oocytes increases the blastocyst rate [15, 16].

Several studies have shown that certain epigenetic markers such as DNA and histone methylation (H3K9 and H3K27) in donor somatic cells are crucial barriers in the epigenetic reprogramming process in SCNT [17]. In this regard, there are several evidences in the literature that the removal of these barriers using DNA methyltransferase inhibitors (DNMTis) and histone methyltransferase inhibitors (HMTis) improves the efficiency of nuclear reprogramming, which may lead to higher quality embryos in terms of pre- and/or post-implantation development in different species [18–20].

Telomere length is one of the most important biomarkers of fertility [21, 22], which is influenced by the in vivo and *in vitro* microenvironment. Telomeres are located at the end of eukaryotic chromosomes and consist of complex proteins and a single-stranded DNA structure with multiple functions [23]. This nucleotide structure, together with a group of proteins, is responsible for chromosome protection and stability. Shiels et al. reported that the telomere length of the first cloned mammal, Dolly, was 20% shorter than that of age-matched sheep obtained by IVF [24]. Although much research has been conducted on telomere elongation in induced pluripotent stem cells [25, 26], there is still insufficient information on the mechanism of telomere elongation in SCNT embryos. The main reason for defective telomere elongation might be inadequate reprogramming. Previous studies have shown that melatonin facilitates telomere elongation in mouse SCNT embryos by inhibiting repressive epigenetic modifications, including DNA methylation and H3K9 trimethylation [22].

Melatonin, a natural hormone, is initially produced and released by the pineal gland. In addition, melatonin is produced in some reproductive tissues such as ovaries, uterus, placenta, and testes. Melatonin is considered a multitasking molecule that regulates many cellular functions, including: antioxidant and anti-apoptotic properties [27–29], induction of autophagy [30, 31], regulation of epigenetic enzymes [5, 22], and telomere length [22, 32].

In the present study, we investigated the effect of melatonin, based on its multifunctional action, on the developmental competence of ovine SCNT embryos. For this purpose, we investigated various indices, including: ROS, glutathione (GSH), apoptotic and autophagic levels, epigenetic status and telomere length in treated and untreated blastocysts.

## Materials and methods

### Ethics statement

All animal experiments and treatments were agreed upon and managed by the Royan Institute Animal Ethics Committee (99000034).

### Chemicals

All chemicals and reagents used in this study were obtained from Sigma-Aldrich Company (St Louis, MO, USA) and Gibco (Life Technologies, Rockville, MD, USA), unless otherwise stated.

### Preparation of melatonin solution

Commercially available melatonin (Sigma, M5250) was dissolved in dimethylsulphoxide (DMSO) (12 mg in 500 μl DMSO), which was formulated to 100 mM stock solution and stored at -20°C. Synthetic oviduct solution (SOF) medium was used to dilute the stock solution to obtain the desired working solution including 0.1 nM, 10 nM and 1 μM based on previous studies. In 1 μM melatonin (the highest concentration of melatonin) the concentration of DMSO is 0.01%. We included control-DMSO (0.01%) group to rule out the effect of DMSO on developmental competence of embryos.

### Oocyte collection and *in vitro* maturation (IVM)

Ovine ovaries were collected from a local slaughterhouse (Fasaran, Isfahan) and transported to the laboratory in flasks containing sterile physiological saline at 20 to 25°C. Cumulus oocyte complexes (COCs) were aspirated from 2–8 mm follicles using an 18-gage needle connected to a vacuum pump. Only oocytes with homogeneous cytoplasm and at least three layers of compact cumulus cells were selected for IVM. The process of IVM of oocytes was according to Hosseini et al. [33]. Briefly, the selected immature COCs were washed in HEPES tissue culture medium 199 (H-TCM) and then placed in TCM medium containing sodium pyruvate, 10%FBS, 10 μg/ml luteinizing hormone (LH; Sigma L5269), 10 μg/ml follicle stimulating hormone (FSH; Sigma F8174), 100 mM 17 β-estradiol (E2; Sigma E4389), 0.1 mM cysteamine (Sigma M9768), 100 ng/ml insulin-like growth factor 1 (IGF1; R&D 291-G1), and 10 ng/ml epidermal growth factor (EGF; Sigma E4127) (maturation medium) [33]. Finally, 10 immature COCs each were transferred to one drop of maturation medium (50 μl) and cultured for 22 h at 38.5°C in a humidified atmosphere containing 5% CO2.

### Sperm preparation and *in vitro* fertilization (IVF) procedure

In this study, IVF-derived embryos were used as controls for the assessment of relative telomere length. The IVF procedure was consistent with our previous studies [33]. For fertilization, frozen semen was thawed (30 s in air and then 45 seconds in 37°C water) and the semen was centrifuged (70 g for 5 min) to remove cryoprotectants. The swim-down method with the PureSperm® gradient was used to isolate motile sperm from immotile sperm. In the next step, 1 × 10^6^/ml motile sperm were added to droplets (50 μl) of fertilization medium containing 10 matured COCs. Isolated motile sperm were co-incubated with matured COCs for 20 h at 38.5°C, 5% CO2 and humidified atmosphere. After 20 h, cumulus cells were removed from the presumptive zygotes by pipetting. Finally, the presumptive zygotes were cultured in SOF medium at 38.5°C, 5% CO_2_, 5% O_2_ and humidified atmosphere. Embryos were collected from different stages of pre-implantation development, including 8–16 cells, morula and blastocyst at day 3, 5 and 7 of development, respectively.

### Donor cell preparation

Ovine ear fibroblast cells (OEFs) were prepared similar to the protocol which has been used in our previous study [34]. The specimen from the skin of ear was taken from a 2 month old female sheep. The presumptive G0 population of donor cells were established by serum starvation (0.5%) for 3–5 days. Fibroblast at passage 3–4 were used for SCNT. Before use, donor cells were trypsinized and resuspended in HTCM-199 plus 0.5% serum.

### SCNT of ovine

After 22 h of *in vitro* maturation, cumulus cells were removed from matured COCs with 300 IU/ml hyaluronidase. After removal, the zona pellucida was removed by treating the mature oocytes with 5 mg/ml pronase for a few seconds. Enucleation of the oocytes was performed manually using a finely pulled Pasteur pipette [34]. To facilitate manual enucleation and reduce injury during oocyte enucleation, zona-free oocytes were treated with 4 μg/ml demecolcin for 1 h at 38.5°C. Then, the cytoplasmic protrusion, including the MII spindle, was removed using a handmade oocyte enucleation pipette. Then, the donor cells were injected into the previtelline space of the enucleated oocytes, and a double electrical pulse of 1000 V for 60 ls was used for oocyte-cell fusion. Then, the activated oocytes were cultured in 6-DMAP drops containing 0, 0.1 nM, 10 nM, and 1 μM melatonin [5, 27, 29, 55] and incubated at 38.5°C, 5% CO2, and maximum humidified atmosphere for 2 h. Finally, 7 embryos were cultured in 20 μl SOFaa drops containing 0, 0.1 nM, 10 nM, and 1 μM melatonin and incubated at 38.5°C, 5% CO2, 5% O2, and humidified atmosphere for 7 days. To prepare the melatonin stock solution, 12 μg of melatonin was dissolved in 500 μl of dimethyl sulfoxide (DMSO) and added to the medium to obtain a final volume of 100 mm. The melatonin-supplemented mSOFaa medium was refreshed at 72 h (on day 3) post- activation. Cleavage and blastocyst rates were determined at day 3 and 7, respectively.

### Intracellular measurement of ROS and GSH in SCNT blastocysts

ROS and GSH level were analyzed at day 7 in blastocysts from the control group and the melatonin-treated group (SCNT-melt (10nM)). To determine the levels of ROS and GSH, 10 mM H2DCFDA (Invitrogen, USA) and 10 mM Cell Tracker™ Blue (CMF2HC, Molecular Probes®, Eugene, OR) were used, respectively. First, blastocysts of each group were washed in phosphate-buffered saline (PBS) containing 1 mg/ml polyvinyl alcohol (PVA) (PBS+PVA) and then incubated in H2DCFDA and Cell Tracker Blue at 38.5°C and kept away from light. Then, the labeled embryos were mounted on glass slides with embedding medium and cover-slips. Embryos were observed under an inverted microscope with appropriate fluorescent filters and analyzed using ImageJ software (version 1.45 s, National Institutes of Health, USA).

### Differential staining

Ovine expanded blastocysts in the control group and the melatonin treated group (10nM) were directly transferred into PBS+PVA medium. Blastocysts were permeabilized with 0.2% Triton X-100 for 20 s and immediately incubated with 30 μg/ml propidium iodide (PI) (Sigma-Aldrich) for 45 s. After extensive washing with HTCM +3 mg/ml bovine serum albumin (BSA), blastocysts were incubated in cold ethanol containing 10 μg/ml Hoechst 33342 (Sigma-Aldrich) for 20 min. Stained embryos were washed in HTCM containing 3 mg/ml BSA, and placed on glass slides, mounted with embedding medium and covered with cover-slip. The slides were examined using an epifluorescence microscope equipped with appropriate fluorescence filters. Images were acquired using DP72 software and analyzed using ImageJ 1.45 V software.

### Immunocytochemistry assay

The derived blastocysts from both groups were washed in PBS+PVA and fixed in 4% paraformaldehyde (PFA) at room temperature (RT) for 30 min. After extensive washing with PBS+PVA, the blastocysts were permeabilized with PBS+PVA and 1% Triton X-100 for 30 min at RT. Subsequently, embryos were washed three times in PBS+PVA and 0.1% Triton X-100 (PBS+PVA-T). For detection of 5-methylcytosine (5mC), blastocysts were treated with 4N HCl for 30 min at RT and then treated with 100 mM Tris-HCl buffer (pH = 8) for 20 min. To avoid non-specific binding of the primary antibodies, blastocysts were blocked with PBS+PVA-T supplemented with 1% BSA and 10% goat serum for 2 h. The embryos were incubated with one of the following antibodies: 5mC antibody (anti-5mC, 1:400; Eurogentec, bi-mecy-0500, Belgium), H3K9ac antibody (dilution 1:200; Sigma, H0913), and LC3B antibody (dilution 1:200; Navusbio, NB100-2220). After washing three times with PBS+PVA-T, embryos were incubated with a matching secondary antibody conjugated to a fluorescent dye; 1:200 dilution of AP124F for 5mC and H3K9ac (Chemicon) and 1:80 dilution of F1262 for LC3B (Sigma) at 37°C for 1 h. Finally, the nuclei of the blastomeres were stained with Hoechst 33342. Finally, immunostained embryos were placed directly on glass slides and mounted with mounting solution and cover-slips. Embryos were observed under epifluorescence microscope with the appropriate fluorescence filters. The relative fluorescence intensity of 5mC, H3K9ac, and LC3B was quantified in the control and melatonin-treated blastocysts using ImageJ software (version 1.45 s, National Institutes of Health, USA).

### Evaluation of mitochondrial function in blastocysts

Mitochondrial membrane potential was determined using the JC1 assay kit (Thermo Fisher Scientific, San Jose, CA, USA) according to the manufacturer’s protocol. In brief, blastocysts were transferred to PBS+PVA containing JC1 at 38.5°C in a humidified atmosphere of 5% CO2 for 10 minutes. Thereafter, the blastocysts were washed several times with PBS+PVA. The ratio of the intensity of red fluorescence (aggregate molecules) to green fluorescence (monomer molecules) was evaluated using ImageJ (National Institutes of Health, Bethesda, MD, USA).

### DNA fragmentation analysis using TUNEL assay

DNA fragmentation index was assessed in the derived blastocysts of the treatment groups using the TUNEL assay kit (Promega, Madison, WI, USA). The TUNEL assay was performed according to the manufacturer’s protocol. In brief, after washing the embryos with PBS-PVA and fixing them with PFA 4%, the blastocysts were treated with a TUNEL mixture (50 μl) containing TdT and dUTP. Then, the blastocysts were counterstained with propidium iodine to label the nucleus. Then, blastocysts were placed on glass slides, covered with coverslips, and observed under a fluorescence microscope (Olympus, Tokyo, Japan).

### Relative telomere length (RTL) determination

Embryos from the IVF, SCNT-Con, and SCNT-Melt (10 nM) groups were collected at different stages (8–16 cells, morula, and blastocyst) and transferred to microfuge tubes. A pool of 20, 15 and 10 embryos for 8–16 cell, morula and blastocyst stages, respectively, in three replications was used for DNA extraction and RTL determination. For each sample, 21 μl of TES buffer (50 mM Tris-HCl, 1 mM Na2EDTA, 20 mM NaCl, and 0.1% SDS (w/v), pH 8.0) and 2.4 μl of proteinase-K (10 mg/ml) were added. After digesting the samples with proteinase-K at 56°C for 1 h, an extension step was performed at 70°C for 10 min. Finally, each sample was diluted 1:2.

After DNA isolation, RTL was determined by a quantitative real-time PCR (qPCR) protocol [35] using the primers for telomere 1 and 2 (Tel 1/2) [36] relative to a reference gene containing one copy ZAR1 [37] (Table 1).

**Table 1:** Primer sequences for telomere length.

Genes	Primer sequences (5’–3’)	Product size(bp)	Annealing temperature (°C)
*Tel1/2*	Forward: 5’-CGGTTTGTTTGGGTTTGGGTTTGGGTTTGGGTTTGGGTT-3’	>76	60
	Reverse: 5’-GGCTTGCCTTACCCTTACCCTTACCCTTACCCTTACCCT -3’		
*ZAR1*	Forward: 5’-AAGTGCCTATGTGTGGTGTG-3’	200	58
	Reverse: 5’-CAGGTGATATCCTCCACTCG-3’		

The following thermocycler profile was used to determine telomere length: 95°C for 30s; 35 cycles of 95°C for 5 s, 60°C for 10 s, and 72°C for 30 s; and a 5-min extension at 72°C. RTL was assessed by the T/S ratio of telomere product amplification (T) to single-copy reference gene (S) [35]. All samples were measured in triplicate, and telomere length was expressed relative to the reference gene.

### Real-time quantitative PCR analysis in ovine SCNT embryos

The effects of the presence or absence of melatonin in the embryo culture medium on the relative expression of candidate genes were examined in the derived blastocysts. The candidate genes were apoptosis-related genes, including *BAX*, *CASPASE-3* and *BCL2*, antioxidant genes including *SOD1* and *GPX4*, developmental related genes including *OCT4*, *NANOG* and *CDX2*, autophagy related genes including (*LC3* and *BECLEN1*) and *GAPDH* (as housekeeping gene).

Total RNA was extracted from SCNT-derived blastocysts (in either SCNT-Con or SCNT-Melt (10 nM)) using an RNeasy micro kit (Exiqon- Qiagen USA) according to the manufacturer’s protocols. For reverse transcription, complementary DNA (cDNA) was prepared using the Biotech rabbit kit (Berlin and Hennigsdorf, Germany). The PCR procedure was performed as follows: Denaturation at 95°C for 30s; 35 cycles of 95°C for 5 s, 60°C for 10 s, and 72°C for 30 s; and a 5-min extension at 72°C. The real-time PCR amplification combination contained l μL of cDNA, 5 L of SYBR Green qPCR Master Mix (Fermentas, Hannover, Germany, MD and USA), 2 μL of ROX, and 0.5 μL of both forward and reverse primers (Table 2) in a total volume of 10 μL. After melting curve analysis, fluorescence values were analyzed using the 2-ΔΔCT formula. Finally, the expression values of the candidate genes were normalized to the expression value of the reference gene.

**Table 2:** Primer sequences for qRT-PCR.

Genes	Primer sequences (5’–3’)	Accession number	Annealing temperature (°C)
*OCT4*	Forward: 5’-AGAAGGGCAAACGATCAAGC -3’	XM_004018968.4	57
	Reverse: 5’-GAATGGGACCGAAGAGTACAGAGT-3’		
*NANOG*	Forward: 5’-GGTGTTTGGTGAACTCTC -3	XM_015094959.2	51
	Reverse: 5’- CATTGATTGTTCCAAGGCT -3		
*GPX4*	Forward: 5’-TCTGTGGAAATGGATGAAAG-3’	XM_027970173.1	54
	Reverse: 5’-AGCCGTTCTTATCAATCAG-3’		
*SOD1*	Forward: 5’-GGAGATAAAGTCGTCGTAAC-3’	NM_001145185.2	54
	Reverse: 5’-TTGTCAGCCTTCACATTG-3’		
*BAX*	Forward: 5’-TGGAGATGAATTGGACAGTAAC-3’	XM_027978594.1	58
	Reverse: 5’-CAAAGTAGAAAAGGGCGACAA-3’		
*CASPASE3*	Forward: 5’-TACACCTCACCACGGAAC-3’	XM_027962551.1	54
	Reverse: 5’-GCCACATTCACTGCTTATACTG-3’		
*CDX2*	Forward: 5’-GCACCATCACCCTCACC-3’	XM_027973894.1	56
	Reverse: 5’-GGGCTTCCGCATCCACT-3’		
*BCL2*	Forward: 5’-AGCATCACGGAGGAGGTAGAC-3’	XM_012103831.3	62
	Reverse: 5’-CTGGATGAGGGGGTGTCTTC-3’		
*LC3B*	Forward: 5’-GCCTTCTTCCTGTTAGTG-3’	XM_004014953.3	54
	Reverse: 5’-AATCCATCTTCATCCTTCTC-3’		
*BECLIN1*	Forward: 5’-CCACTGAGAGGACAAGAAG-3’	XM_004012945.4	54
	Reverse: 5’-AGAGCAGAGCAAGCATTC-3’		
*GAPDH*	Forward: 5’-GTTCAACGGCACAGTCAAG-3’	NM_001190390.1	60
	Reverse: 5’-TACTCAGCACCAGCATCAC-3’		

### Statistical analysis

All assessments were carried out at least three times. Data is presented as mean ± S.E.M. Statistical significance was set at *P* < 0.05. One-way analyses of variance (ANOVA) was applied to study the effect of melatonin on cleavage and blastocyst rates (α = 0.05) followed by Tukey post-hoc test. In addition, differences between mean relative telomere lengths at the different embryonic stages in various groups (IVF vs. SCNT-Con and SCNT-Melt (10 nM)) were compared by One-way ANOVA. The remaining assessments were analyzed by independent samples t-test. GraphPad Prism (v.6.0.1) and IBM SPSS program (v.23, NY, USA) were used for creating graphs and statistical analysis, respectively.

## Results

### The optimized concentration of melatonin during *in vitro* culture (IVC) accelerates the blastocyst formation rate in ovine SCNT embryos

First, we investigated whether supplementation of embryo culture medium with melatonin improved cleavage and blastocyst rates of ovine SCNT embryos. Immediately after chemical activation, reconstituted SCNT oocytes were cultured in SOF medium supplemented with 0, 0.1 nM, 10 nM, and 1 μM melatonin during IVC (seven days). According to the half-life of melatonin, the culture medium was refreshed 72-h post activation. Cleavage and blastocyst rates were assessed at day 3 and 7 of in vitro development [33]. Our results showed that treatment with different concentrations of melatonin did not affect the cleavage rate at day 3 of IVC (*P* > 0.05) (Table 3). Treatment with 0.1 nM and 1 μM melatonin did not accelerate the blastocyst yield compared with the control group in ovine SCNT embryos (*P* > 0.05), whereas 10 nM melatonin significantly increased the blastocyst rate of ovine SCNT embryos (*P* < 0.05) (Table 3). So, based on the concentration optimization results, we used 10 nM melatonin for the further assessment throughout the study.

**Table 3:** Evaluation of various concentrations of melatonin on developmental competence of ovine SCNT embryos.

Group	No. of embryos	No. of cleaved embryos (Mean ± S.E.M. %)	No. of blastocysts (Mean ± S.E.M. %)
Control	258	236 (95.34 ±2.37) ^a^	31 (12.85 ± 3.90) ^b^
Control DMSO (0.01%)	143	116 (93.14 ±5.51) ^a^	13 (10.63 ± 1.24) ^b^
0.1 nM melatonin	210	178 (91.44 ± 5.39) ^a^	20 (10.80 ± 2.48) ^b^
10 nM melatonin	224	166 (87.11 ± 1.79) ^a^	47 (28.74 ± 4.61) ^a^
1μM melatonin	198	163 (85.73 ± 0.68) ^a^	11 (6.85 ± 1.76) ^b^

At least five replications were performed for each treatment. Developmental rates of treated embryos were monitored as cleavage and blastocyst rates at day 3 and 7, respectively. Within a column, developmental rates with different superscripts (a and b) are significantly different from each other (P< 0.05).

### Effects of melatonin on the quality of SCNT derived blastocysts

**Fig 1:**
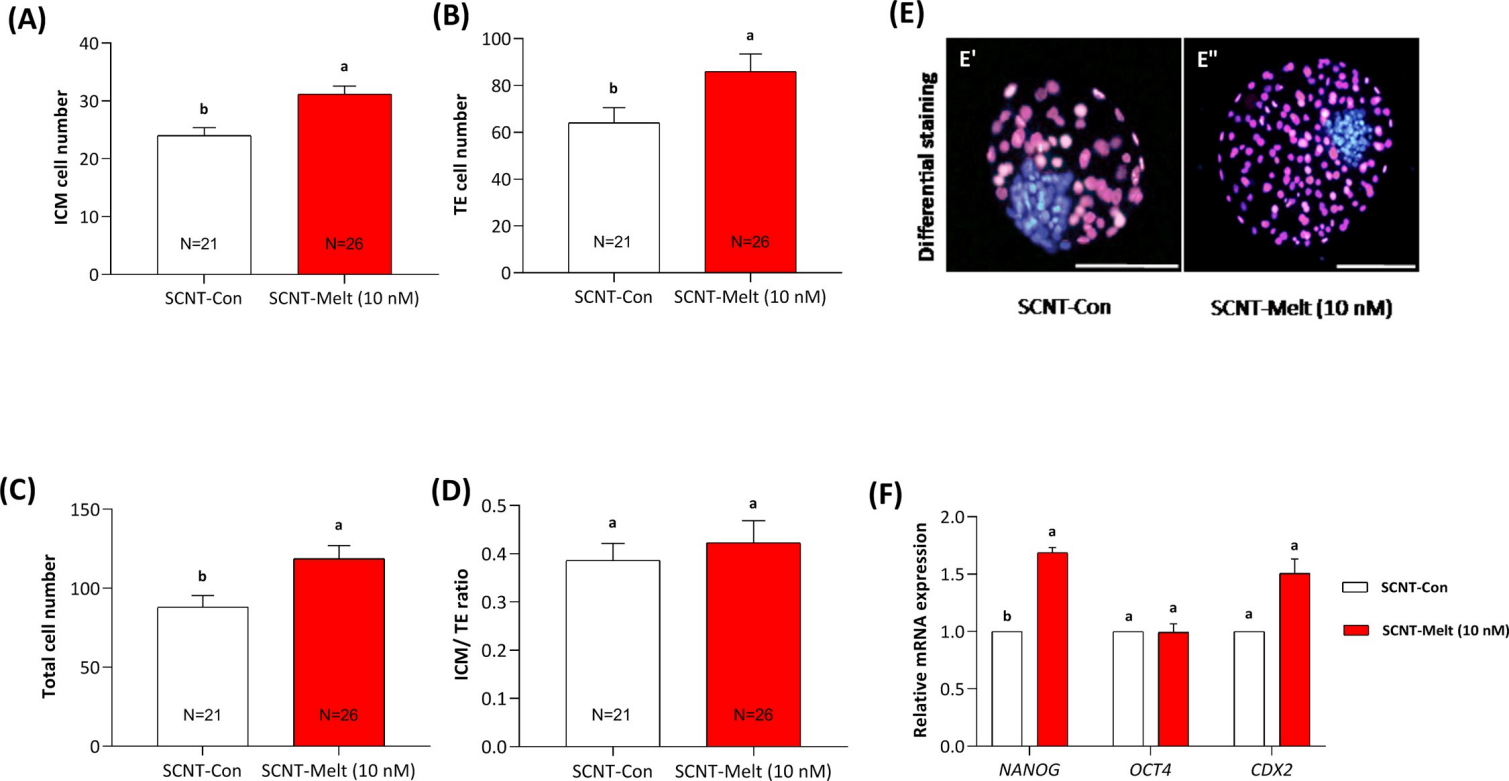
The effect of melatonin on the quality of ovine SCNT blastocyst embryos. The number of A) ICM, B) TE, C) TCN and D) ICM/ TE ratio that was assessed by differential staining of derived blastocysts. E) Representative images from differential staining of blastocysts derived from control and melatonin-treated groups. Scale bar, 100μm F) The relative mRNA expression level of pluripotency and trophectodermal genes (*OCT4*, *NANOG* and *CDX2*) in SCNT blastocysts from SCNT-Melt (10 nM) and SCNT-Con groups. The data were shown as mean ± SEM. Values with different letters have a statistically significant difference (*P*<0.05).

For evaluating the consequence of melatonin on quality of derived blastocysts, differential staining was carried out to determine the number of ICM, TE, and TCN cells. As depicted in Fig 1A–1C, our results showed that the number of, ICM, TE, and TCN in blastocysts treated with SCNT-Melt (10 nM) increased significantly compared to the SCNT-Con (*P* <0.05) (Fig 1A–1C). However, no significant difference was observed in the ICM/ TE ratio between treatment groups (*P*> 0.05, Fig 1D).

To further analyze the quality of derived blastocyst, the relative expression of developmental related genes including *OCT4* and *NANOG* (pluripotency genes) and *CDX2* (trophectoderm gene) (Fig 1F). The results showed that the relative mRNA expression of *NANOG* increased significantly in the SCNT-Melt (10 nM) group compared to the SCNT-Con (*P* <0.05). In contrast, the relative mRNA expression of *OCT4* and *CDX2* genes was not different between SCNT-Melt (10 nM) and their counterparts in SCNT-Con group (*P* >0.05).

### Effect of melatonin on ROS level of ovine SCNT embryos

**Fig 2:**
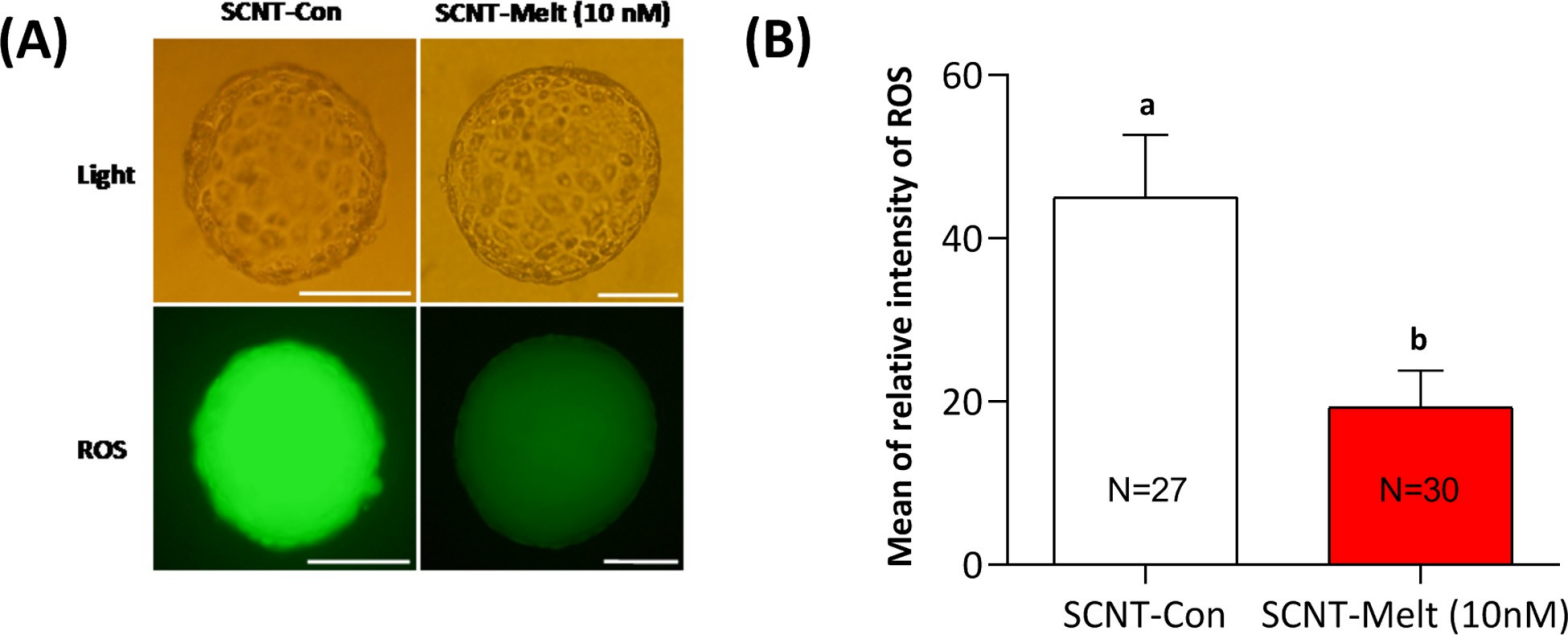
Effect of melatonin on ROS levels in ovine SCNT embryos at blastocyst stage. A) Representative images from H2DCFDA staining of blastocysts derived from SCNT-Con and SCNT-Melt (10 nM) groups. Scale bar, 100μm. B) The mean of relative fluorescent intensity of ROS in ovine SCNT blastocyst. The data were shown as mean ± SEM. Values with different letters have a statistically significant difference (*P*<0.05).

Analysis showed that melatonin decreased the level of ROS, as measured by relative fluorescence intensity, in blastocysts treated with SCNT-Melt (10 nM) compared with blastocysts in the SCNT-Con group (P < 0.05) (Fig 2A and 2B).

### Effect of melatonin on GSH level of ovine SCNT embryos and gene expression of regulating enzymes

**Fig 3:**
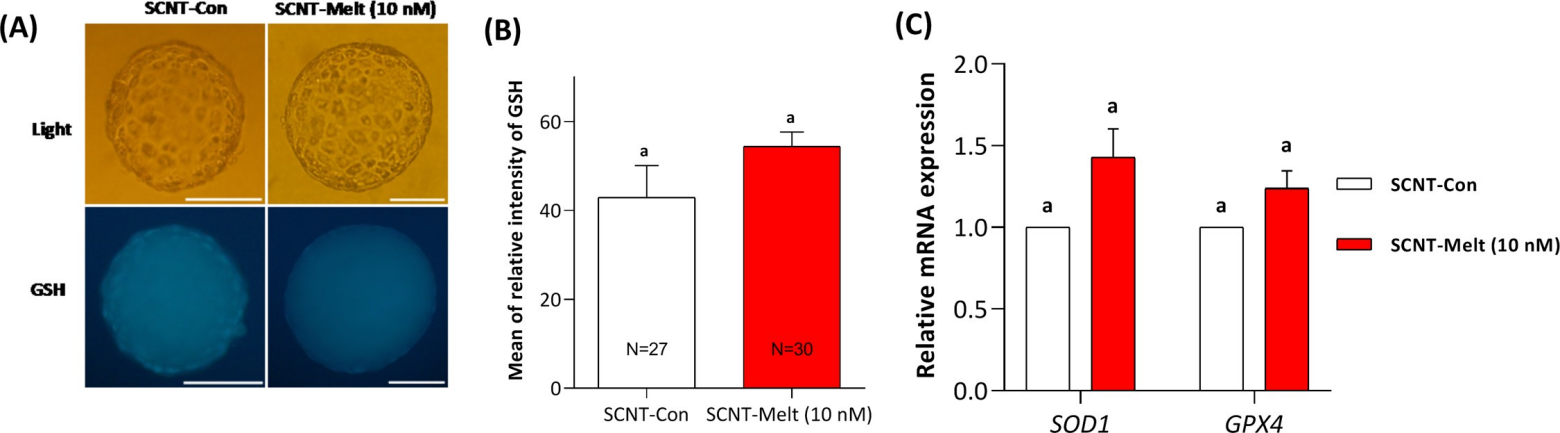
Effect of melatonin on GSH level of ovine SCNT blastocysts and the relative mRNA expression of regulating enzymes. A) Fluorescent images of glutathione content in ovine SCNT blastocysts in control and melatonin-treated groups assessed by Cell Tracker Blue. Scale bar, 100μm B) The mean of relative fluorescent intensity of GSH in ovine SCNT blastocyst. C) The relative mRNA expression level of antioxidant genes (*SOD*1 and *GPX4*) in SCNT-Melt (10 nM) and SCNT-Con groups. The data were shown as mean ± SEM. Values with different letters have a statistically significant difference (*P*<0.05).

In addition to ROS, we examined the effect of melatonin during IVC on GSH levels and associated regulatory enzymes. As shown in Fig 3A and 3B, supplementation of IVC medium with SCNT-Melt (10 nM) did not alter GSH levels in ovine SCNT blastocyst embryos (P < 0.05, Fig 3A and 3B). In addition, our results showed that the relative mRNA expression of *SOD1* and *GPX4* did not change in the SCNT-Melt (10 nM) group compared with the SCNT-Con group (P < 0.05, Fig 3C).

### Effect of melatonin on apoptotic index of ovine SCNT blastocysts and gene expression of regulating enzymes

**Fig 4:**
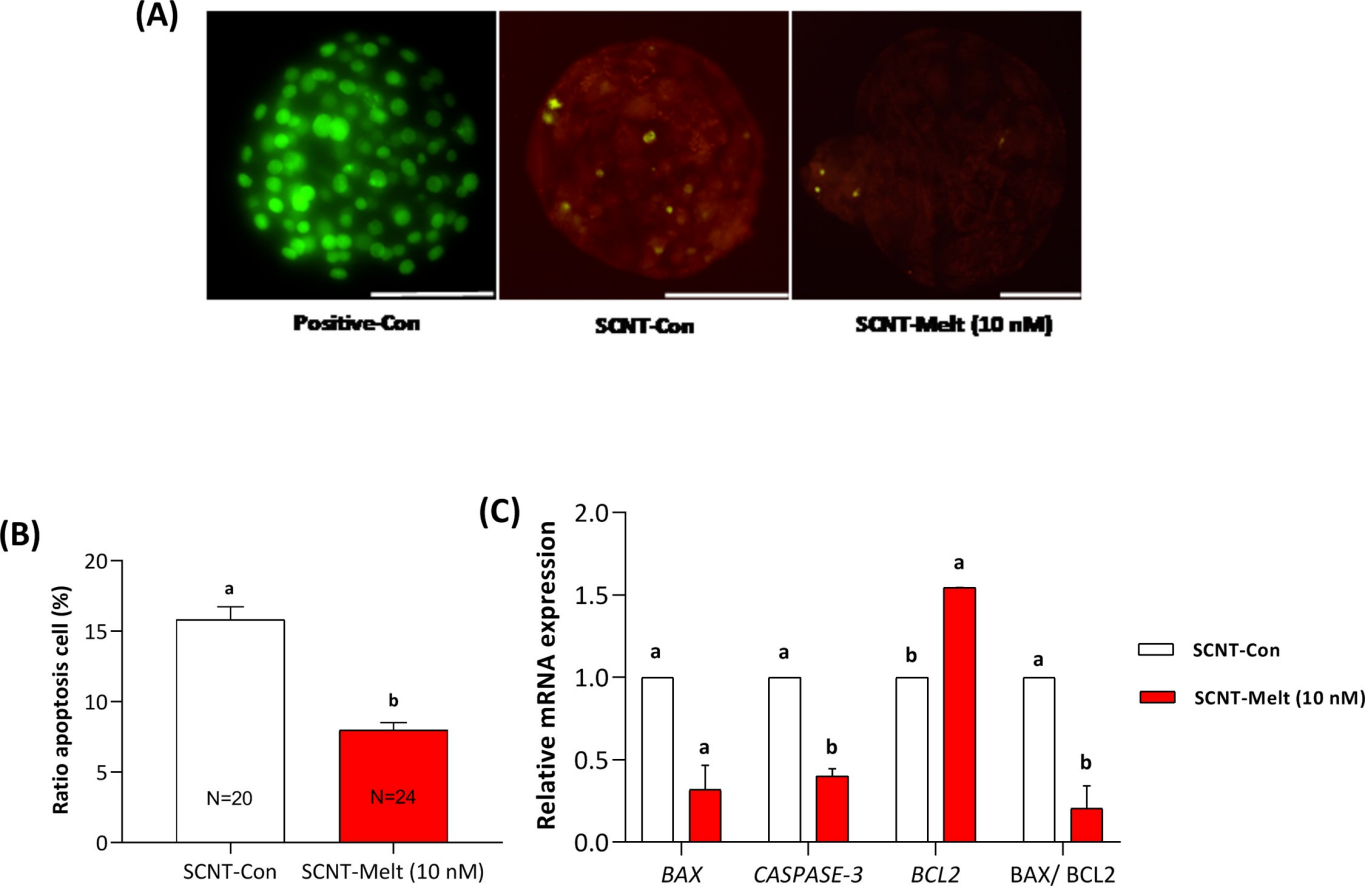
Effect of melatonin on apoptotic index of ovine SCNT embryos and relative mRNA expression of regulating enzymes. A) TUNEL assay of blastocysts (green). Each sample was counterstained with DAPI to visualize DNA (red). Scale bar, 200μm B) Total number of apoptotic cells in derived blastocyst from treated groups. C) The relative expression level of apoptosis genes (*CASPASE3*, *BAX* and *BCL2*) in SCNT-Melt (10 nM) and SCNT-Con groups. The data were shown as mean ± SEM. Values with different letters have a statistically significant difference (*P* <0.05).

As depicted in Fig 4A and 4B, total number of apoptotic cells was remarkably lower in SCNT-Melt (10 nM) group as compared to the SCNT-Con group (*P* < 0.05).

The relative mRNA expression of anti-apoptotic (*BCL2*) and pro-apoptotic (*BAX* and *CASPASE-3*) genes in ovine SCNT blastocysts was evaluated by real-time RT-PCR. Our results demonstrated that the relative mRNA expression of *BCL2* in SCNT-Melt (10 nM) group was significantly up-regulated as compared to the SCNT-Con group (*P* <0.05). It was also observed that the relative mRNA expression of *CASPASE-3* in blastocysts treated with SCNT-Melt (10 nM) decreased compared to the SCNT- Con group (*P* <0.05). However, the relative mRNA expression of *BAX* in the SCNT-Melt (10 nM) group did not differ with the SCNT- Con group (*P* <0.05) (Fig 4C). Furthermore, the BAX/BCL2 ratio as a marker of apoptosis was decreased in SCNT-Melt (10 nM) group compared to SCNT- Con group (*P*< 0.05) (Fig 4C).

### Effect of melatonin on the autophagy status of derived blastocysts

**Fig 5:**
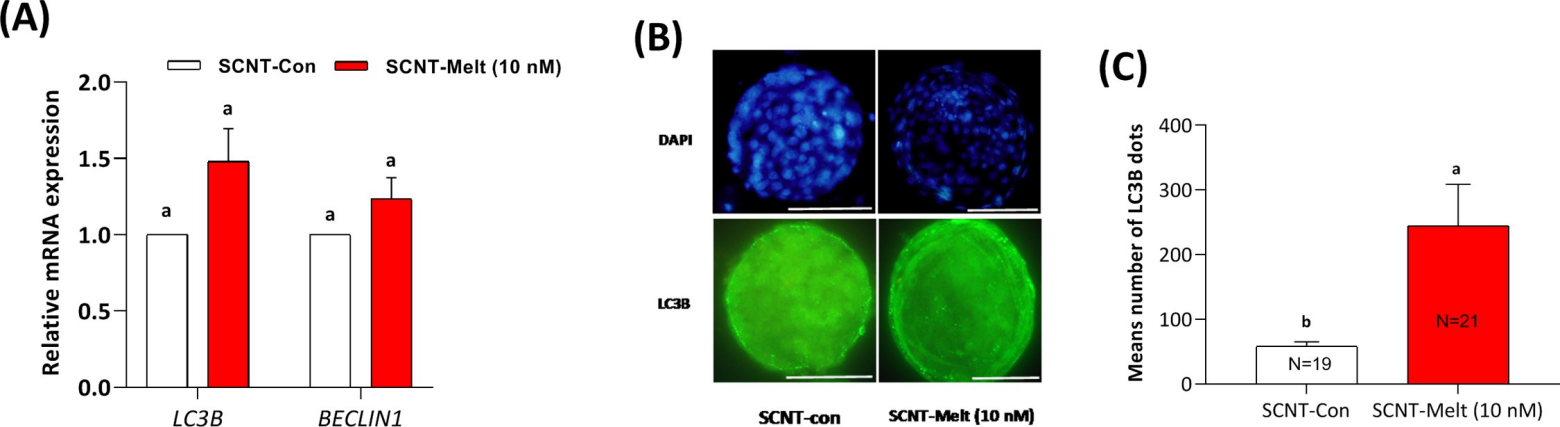
Effect of melatonin on the autophagy status of derived blastocysts. A) The relative expression levels of autophagy genes (*LC3B* and *BECLINE1*) in SCNT-Melt (10 nM) and SCNT-Con groups. B) Representative images of LC3B protein dots in ovine SCNT embryos. Scale bar, 100μm C) The mean number of LC3B dot in ovine SCNT embryos treated with 10 nM melatonin compared to control group. The data were shown as mean ± SEM. Values with different letters have a statistically significant difference (P <0.05).

In this study, to assay whether melatonin affects autophagy status in ovine SCNT blastocysts, we evaluated the relative mRNA expression of important autophagy genes (*LC3B* and *BECLINE1*). Our data revealed that in embryos treated with SCNT-Melt (10 nM) the relative mRNA expression of *LC3B* and *BECLINE1* did not change compared to the SCNT-Con group (*P* <0.05) (Fig 5A).

In addition, we assessed the level of LC3B protein in derived blastocysts by immunocytochemistry assay. As depicted in Fig 5B and 5C, the mean number *LC3B* dots in blastocysts treated with SCNT-Melt (10 nM) significantly increased as compared to SCNT-Con group (*P* <0.05).

### Effect of melatonin on epigenetic marks 5mC and H3K9ac in ovine SCNT embryos

**Fig 6:**
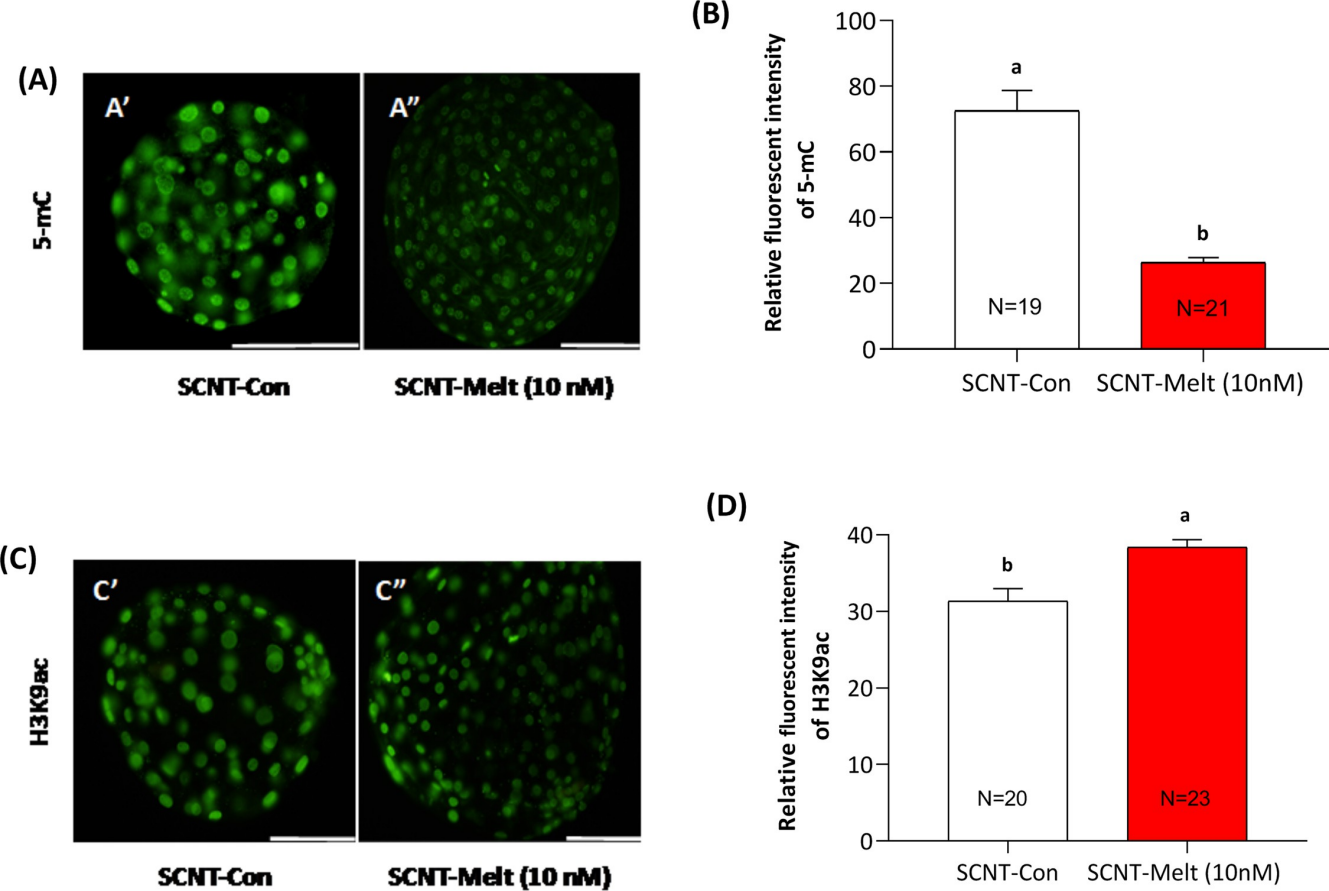
Global level of 5mC and of H3K9ac in ovine SCNT embryos. (A) Staining of 5mC in embryos in the SCNT, and SCNT-Melt (10 nM) groups at blastocyst stages. Scale bar, 100μm (B) Quantification of 5mC signal intensities in embryos in the SCNT, and SCNT-Melt (10 nM) groups at blastocyst stages. (C) Staining of H3K9ac in embryos in the SCNT, and SCNT-Melt (10 nM) groups at blastocyst stages. Scale bar, 100μm (D) Quantification of H3K9ac signal intensities in embryos in the SCNT, and SCNT-Melt (10 nM) groups at blastocyst stages. The data were shown as mean ± SEM. Values with different letters have a statistically significant difference (*P*<0.05). The experiments were performed more of three times. In each replication, n = 12–20 per group. SCNT, somatic cell nuclear transfer.

In the next step, we investigated the epigenetic modifying effect of melatonin in terms of global DNA methylation (5-mC) and H3K9ac levels in ovine SCNT derived blastocysts stage in both control and melatonin-treated groups. As depicted in Fig 6, the 5mC level in blastocysts treated with SCNT-Melt (10 nM) significantly decreased as compared to the SCNT-Con group (*P*< 0.05, Fig 6A and 6B). Also, we observed that the global H3K9ac level in ovine SCNT blastocysts was increased in SCNT-Melt (10 nM) as compared to SCNT-Con group (*P*< 0.05, Fig 6C and 6D).

### Effect of melatonin on the mitochondrial membrane potential (ΔΨm)

**Fig 7:**
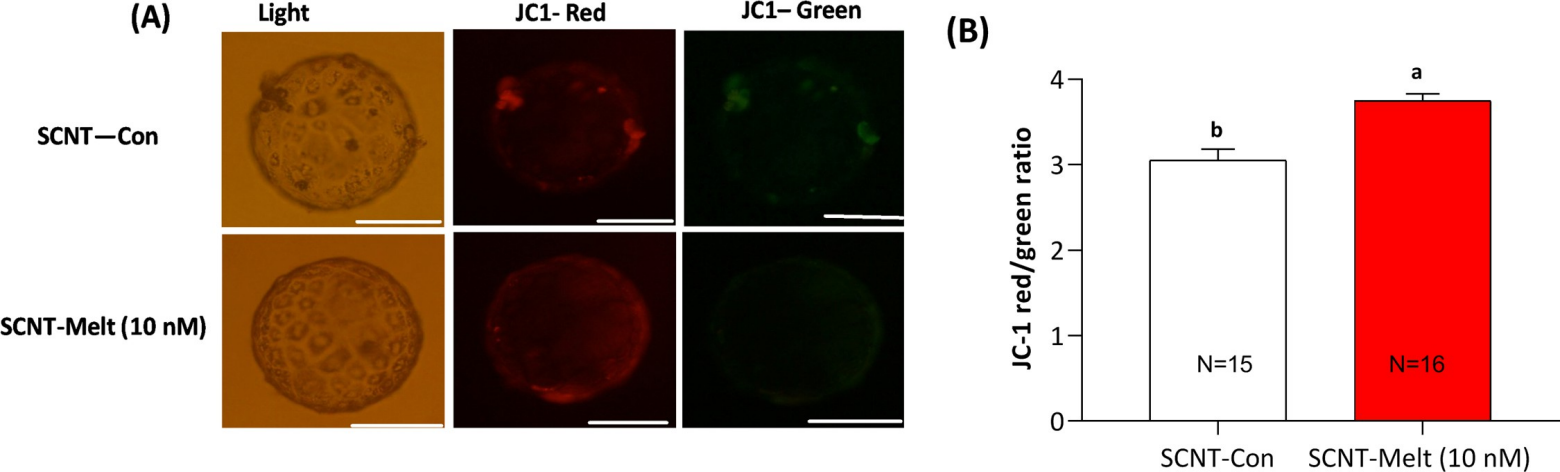
Effect of melatonin on the mitochondrial membrane potential (ΔΨm) in ovine SCNT blastocysts. (A) Relative fluorescent intensity of mitochondrial membrane potential in ovine SCNT blastocysts. Scale bar, 100μm (B) Quantity of mitochondrial membrane potential in ovine SCNT embryos treated with 10 nM melatonin or control group. The data were shown as mean ± SEM. Values with different letters have a statistically significant difference (*P*<0.05).

For evaluation the effect of melatonin on mitochondrial membrane potential of embryos, derived blastocysts were labeled with JC-1 and the intensity of red fluorescent to green fluorescent was assessed. As depicted in Fig 7A and 7B, the red/green ration (the level of ΔΨm) in SCNT-Melt (10 nM) was increased significantly as compared to SCNT-Con group (*P*<0.05).

### Effects of melatonin on the relative telomere length of SCNT derived blastocysts

**Fig 8:**
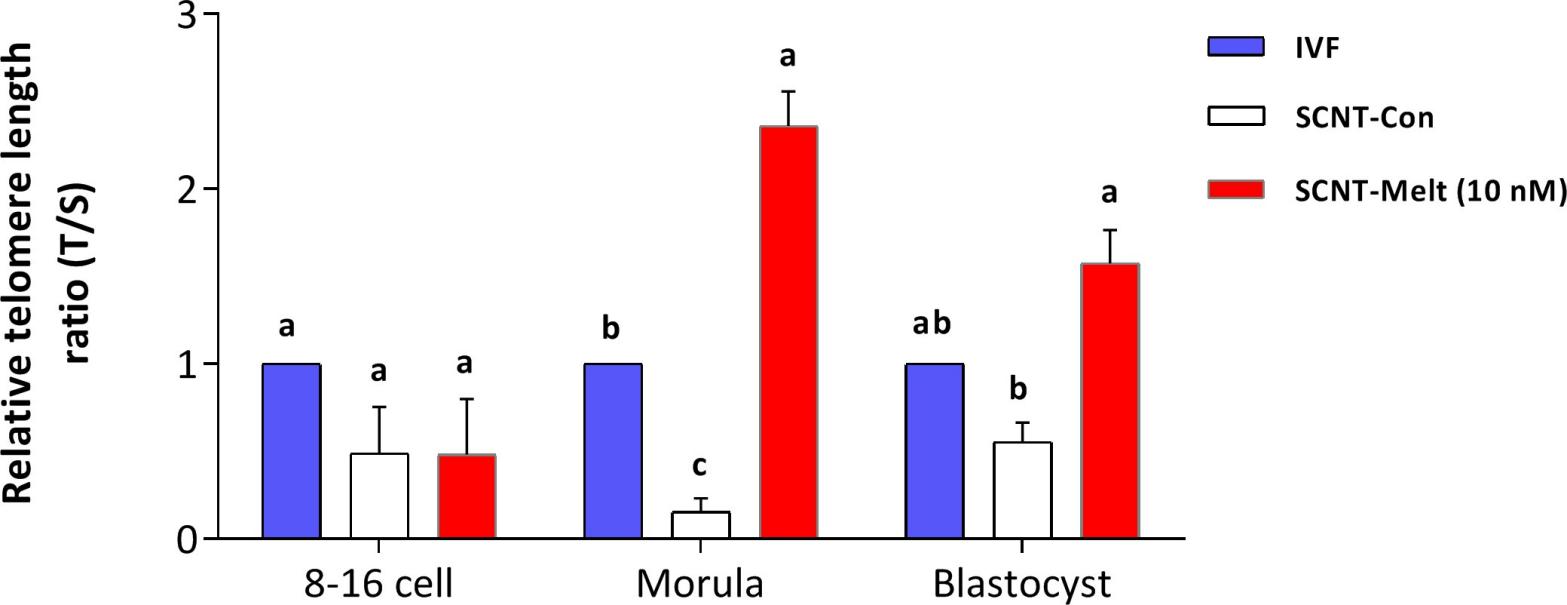
Effects of melatonin on the relative telomere length of SCNT derived blastocysts. The relative telomere length between SCNT-Melt (10 nM) and SCNT-Con embryos at 3 distinct developmental stages (8-cell, morula and blastocyst) was evaluated by RT-qPCR. The telomere length of IVF-derived embryos was evaluated as standard group. The data were shown as mean ± SEM. Values with different letters have a statistically significant difference (*P*<0.05).

The relative telomere length between melatonin-treated and control SCNT embryos at 3 different developmental stages (8-cell, morula, and blastocyst) was examined by RT -qPCR. The telomere length of IVF embryos was evaluated as a control group. As shown in Fig 8, the relative telomere length at the 8–16 cell stage was similar in all groups (*P* > 0.05, Fig 8). At the morula stage, the relative telomere length was significantly shorter in the SCNT-Con group compared with the IVF embryos. Interestingly, we found that the morula from the SCNT-Melt (10 nM) group had longer telomere length than the morula from the SCNT-Con and IVF groups (P < 0.05, Fig 8). At the blastocyst stage, the relative telomere length was significantly increased in the SCNT-Melt (10 nM) group compared with the SCNT-Con group (*P* < 0.05, Fig 8). Finally, there was no significant difference in the relative telomere length of IVF-derived blastocysts in either the SCNT-Melt (10 nM) group or the SCNT-Con group (*P* < 0.05).

## Discussion

It is well known that most SCNT embryos suffer from various cellular and molecular defects, including mitochondrial dysfunction, abnormal epigenetic modifications, altered gene expression, and defects in telomere elongation. In this study, we hypothesized that melatonin, through its multitasking function, could mitigate the above defects in SCNT embryos and increase the developmental potential of SCNT embryos.

SCNT-derived embryos are exposed to higher hyperoxic environment compare to other in vitro-derived embryos due to various manipulations during the SCNT procedure (including serum starvation of donor cells, enucleation, electrofusion, artificial activation) and also due to defects in endogenous antioxidant defenses [38, 39].

Hyperoxic environment in embryos leads to an accumulation of excessive ROS, which may result in cellular and molecular impairments in pre-implantation embryos, leading to embryonic developmental defects and growth retardation [40]. In addition, exposure of a well-differentiated cell (fibroblast) to ooplasm with high reprogramming potential may also induce cellular stress and be responsible for some of these defects [14]. To increase cellular resistance to oxidative stress in embryos and reduce oxidative stress status in the microenvironment, the most effective strategy is to supplement culture media with exogenous antioxidants. Melatonin has been shown to have anti-inflammatory and antioxidant properties in the body by scavenging ROS and nitrogen species [41]. We demonstrated that supplementation of IVC medium with melatonin (10 nM) significantly reduced the level of ROS in the derived blastocysts (Fig 2).

It is worth noting that despite the reduction in ROS level, neither GSH level nor mRNA expression of its regulating enzymes, including SOD1 and GPX4, were altered in the embryos (Fig 3). These results suggest that melatonin has no effect on the level of reducing peroxidation. Therefore, it is likely that melatonin exerts its effect via lowering the content of ROS, which in turn improves the reprogramming efficiency of sheep SCNT embryos.

Mitochondria play an important role during early embryonic development. Several studies have shown that mitochondrial dysfunction is associated with various defects in oocyte and embryo development [42]. Mitochondrial membrane potential is an indicator of mitochondrial function and cellular viability in embryos [43]. In this study, we demonstrated an improvement in mitochondrial function in treated embryos. The potential mechanism for melatonin as a broad-spectrum antioxidant is probably to lower the ROS level without altering the GSH level, leading to an improvement in mitochondrial function. All these consequences of treating SCNT embryos with melatonin resulted in higher developmental competence of SCNT embryos. However, a high level of melatonin (1 μM) had a negative effect on the blastocyst formation rate, possibly indicating that a low level of ROS is necessary for proper cell function and embryonic development (Table 3).

In agreement with previous reports [5, 44], our results showed that the quality of blastocysts in SCNT-Melt (10 nM) was improved in terms of the number of ICM, TE and TCN. To clarify the main mechanism related to this result, the relative mRNA expression of three developmental factors was examined, and we found that melatonin increased the expression of *NANOG* (P < 0.05) and *CDX2* (P > 0.05) (Fig 1F). As a main regulator of pluripotency loop, *NANOG* and *CDX2* are imperative for preserving the ICM cell and TE, respectively. Thus, the difference is likely due to higher expression of *NANOG* and *CDX2* in the melatonin-treated SCNT blastocysts.

DNA fragmentation index is another indicator of blastocyst quality [45] and an important factor in embryo development [45]. In this study, to evaluate the relationship between apoptosis and decreased ROS levels, we examined the level of apoptotic cells and the expression of apoptotic genes in the presence or absence of melatonin. Our results showed that 10nM melatonin in embryo culture medium significantly reduced apoptosis in ovine SCNT embryos (Fig 4A and 4B). Taken together, these results suggest that melatonin can improve the quality of derived blastocysts by lowering the DNA fragmentation index. Similarly, melatonin treatment has been shown to reduce apoptosis in SCNT embryos from cattle [5], pigs [27], and mice [29].

In addition, melatonin was shown to downregulate the expression of the pro-apoptotic gene *CASPASE-3* and upregulate the anti-apoptotic gene *BCL2* (Fig 4C). The effects of melatonin on the relative expression of genes could lead to reduced apoptosis of cells in melatonin-treated blastocyst embryos. These results are consistent with previous studies performed in bovine IVF embryos [46] and porcine SCNT embryos [5, 44].

In this study, to determine whether the decrease in apoptosis rate was due to the induction of autophagy, we examined the relative mRNA expression and protein levels of autophagy-related genes in SCNT blastocysts treated with melatonin. Our results showed that supplementation of IVC medium with melatonin increased LC3 dots in the derived blastocysts without altering mRNA gene expression of *BECLIN1* and *LC3* (Fig 5C).

Consistent with our results, Zhang et al. reported that melatonin increased *LC3* expression and decreased *BAX* expression, suggesting that autophagy and apoptosis were stimulated and inhibited, respectively, in the presence of melatonin [30]. These results suggest that autophagy plays an important role in SCNT embryo development and that inhibition of autophagy may be one of the main reasons for the low developmental efficiency of SCNT embryos.

Epigenetic marks regulate gene expression and stabilize genome integrity. The association between low efficiency in mammalian SCNT and abnormal epigenetic reprogramming is a fact that has been recognized by several scientists in different species [17, 47, 48]. Our results showed that the global H3K9ac level was higher in SCNT-Melt (10 nM) blastocysts than in the SCNT-Con group (Fig 6C and 6D). This observation is consistent with the reports of Su et al. [5]. Histone hyperacetylation facilitates the access of various transcription factors to the nucleosome and alleviates transcriptional repression [49, 50], which in our case enhanced nuclear reprogramming of SCNT embryos and promoted in vitro embryo development [51]. Consistent with histone hyperacetylation (H3K9ac), we observed that the 5mC level was lower in SCNT embryos treated with melatonin (Fig 6A and 6B). It is known that melatonin, as a lipophilic small molecule, can easily cross the cell membrane, accumulate in the nucleus, and interact with specific nuclear binding sites. Binding to specific nuclear binding sites can inhibit the action of DNMTs and decrease the level of DNA methylation [52]. Therefore, it is possible that melatonin supplementation, by lowering the DNA methylation level in SCNT embryos, promotes nuclear reprogramming and improves both the quantity and quality of the resulting embryos [53–55].

Among other defects in SCNT embryos, several studies have shown that telomere length is not efficiently reset by SCNT [56]. Nevertheless, the relationship between increased telomere length and SCNT reprogramming competence is unclear, especially at the pre-implantation stage of embryonic development. Despite many efforts, SCNT embryo competence is low in most species, and the mechanism of telomere reprogramming after SCNT in various mammalian species is also still unknown [3, 48, 57]. In this context, increasing the telomere length in SCNT embryos could help to improve the efficiency of SCNT. In this study, we demonstrated that supplementing the culture medium with melatonin increased relative telomere length compared to IVF and SCNT embryos at the morula and blastocyst stages, although the difference was significant only at the morula stage (Fig 8). Thus, we demonstrated that melatonin can increase relative telomere length during SCNT reprogramming in morula and blastocyst embryos (Fig 8). In this context, Young et al. reported that melatonin facilitates telomere rejuvenation in mouse SCNT embryos [22]. This improvement in telomere length may be associated with an improvement in the oxidative microenvironment in the culture medium of the embryos. Therefore, melatonin could postpone telomere shortening by reducing oxidative stress in the ovine SCNT blastocysts.

## Conclusion

The results showed that melatonin was able to accelerate the reprogramming of telomere length in ovine SCNT embryos, in addition to its various beneficial effects such as increasing antioxidant capacity, reducing DNA damage, and improving the quality of derived blastocysts, all of which resulted in a higher developmental rate under *in vitro* conditions. However, the evaluation of the effect of melatonin on *in vivo* development in ovine species is of great interest and can be pursued in our future studies.

## Supporting information

S1 Fig Effects of melatonin on the relative telomere length of SCNT derived blastocysts. The relative telomere length between IVF, SCNT-Melt (10 nM) and SCNT-Con embryos at 3 distinct developmental stages (8-cell, morula and blastocyst) was evaluated by RT-qPCR. The telomere length of fibroblast cells was evaluated as control group. The data were shown as mean ± SEM. Values with different letters have a statistically significant difference (*P*<0.05).
(TIF)

## Abbreviations

DNMTis: DNA methyltransferases inhibitors
GSH: Glutathione
HMTis: histone methyltransferase inhibitors
ICM: inner cell mass
IVF: *in vitro* fertilization
IVM: *in* vitro maturation
ROS: reactive oxygen species
SCNT: Somatic cell nuclear transfer
TCN: total cells number
TE: trophectoderm
