# Supplementary figures and images for: Melatonin accelerates the developmental competence and telomere elongation in ovine SCNT embryos
Source: PLoS One. 2022 Jul 21;17(7):e0267598. doi: 10.1371/journal.pone.0267598 (PMC9302776; doi:10.1371/journal.pone.0267598)

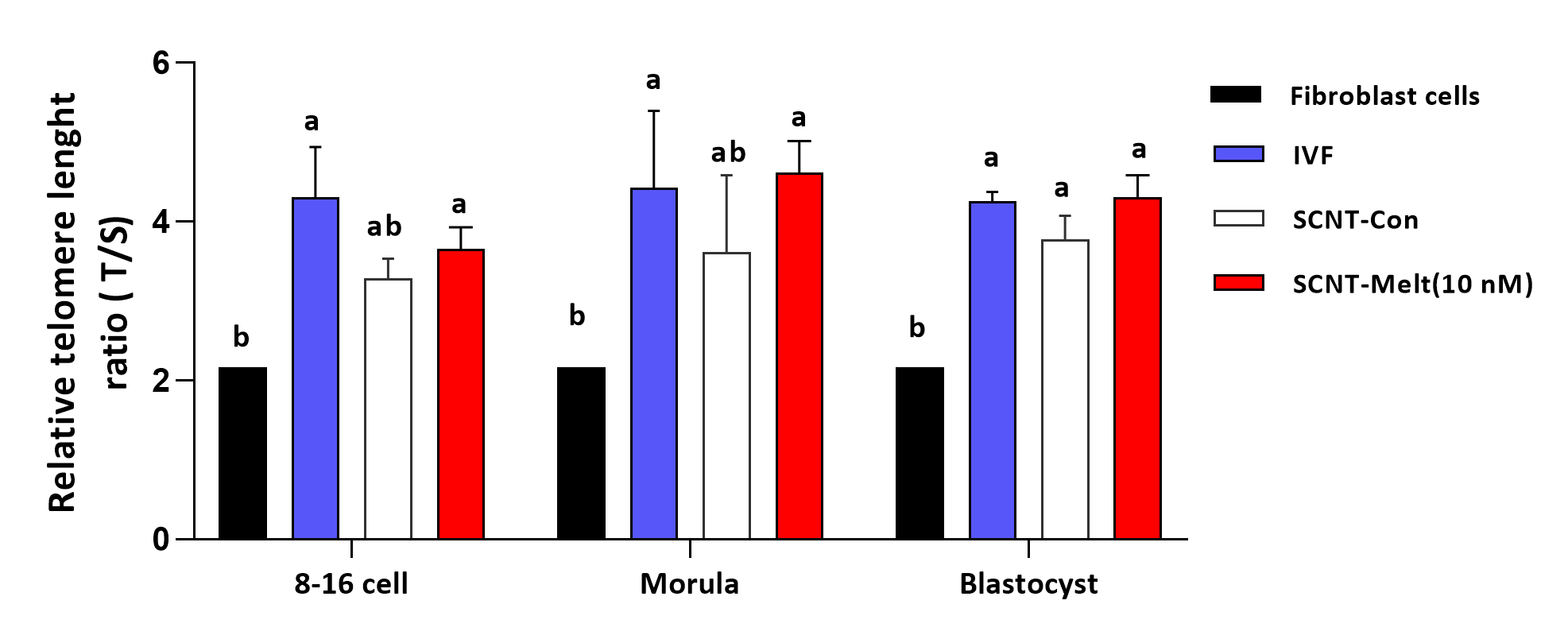

Supplement: S1 Fig — The relative telomere length between IVF, SCNT-Melt (10 nM) and SCNT-Con embryos at 3 distinct developmental stages (8-cell, morula and blastocyst) was evaluated by RT-qPCR. The telomere length of fibroblast cells was evaluated as control group. The data were shown as mean ± SEM. Values with different letters have a statistically significant difference (P<0.05). (TIF) [file pone.0267598.s001.tif]
